# Progress in Psychiatry

**Published:** 1900-10-13

**Authors:** 


### PROGRESS IN PSYCHIATRY.

(*Continued from page* 386, vol. xxviii.)

**Toxic Action of Morphia.**—Morphia causes insanity more rarely than the other toxic substances which are indulged in by mankind for purposes of pleasure and for experiencing temporary feelings of well-being (euphoria). Its prolonged abuse gives rise to psychical disorders (disorders of personality) that confine themselves to the "domain of semi-alienation" (Regis)—the borderland between sanity and insanity. Opium seems to be the intoxicant which, next to alcohol, has been indulged in most largely by the human race. Its abuse as a narcotic is traceable to early historic times. Murrell has shown that the inhabitants of the fens of Lincolnshire had long employed opium as a prophylactic against malaria. The same practice (according to Talbot [58]) prevailed in certain malarial regions of New Jersey and Pennsylvania, where the use of strong infusion of poppy was common. A tendency to opium-tolerance appears to have been observed in the children of parents, especially mothers, addicted to the opium habit. The fœtus *in utero* receives the toxic agent through the placenta, just as it is known to receive alcohol from an inebriated mother. In consequence, the child in the first months of infancy must be nourished on the milk of an opium-using woman, or given opium in some other way, lest it perish.[59] To this fact Calkins [60] was the first to call attention, and his results were confirmed by Erlenmeyer of Berlin, Mattison of Brooklyn, Kiernan [61] of Chicago, and others. The last-mentioned author, after quoting numerous instances of the congenital opium-habit where opium was needed to preserve the infant during the first months of life, points out that pigeons whose ancestors were fed on poppies became tolerant of opium. Murrell found that the same was relatively true of persons descended from ancestors (in Bedfordshire) who used infusions of poppy as a prophylactic against malaria. It appears, moreover, that the children of parents addicted to morphia were often still-born or were the victims of some form of cerebral and mental defect, with a marked tendency to infantile convulsions (Amabile), or to congenital idiocy and imbecility and other forms of cerebral degeneration. Levinstein has shown by experiments on pregnant bitches and rabbits that the use of opium during pregnancy produced either abortion or still-births, or rapidly-dying offspring. These facts regarding the far-reaching deleterious effects of opium, both on the parent and the offspring, suffice to show its resemblance to alcohol as a toxic agent (Hospital, December 30th, 1899, pp. 211-12).

The opium-eater and the morphia *habitué* indulge in these drugs for the same reason, viz.: to experience pleasure and euphoria in the first instance. Agreeable illusions and hallucinations of an erotic nature are attributed to opium, but these do not persist in the later stages of the opium-habit. The mental conditions induced are more subtle, extraordinary, delusional, phantasmagorial, semi-delirious and megalo-maniacal. They have been minutely and aptly described by the opium-eater De Quincey.[62] Ideas of space and time are incredibly magnified or set at naught by the wonderful illusions and delusions attending opium-intoxication. Extraordinary transformations of personality are experienced by the subject. He is at one time a priest, then a wooden idol, and at the next moment a victim for sacrifice on the altar. He is an old man, a woman, a winged being, or even the Almighty himself. He "assists" at sacrifices and rites, at funerals, at the Day of Judgment itself. He lives for centuries in temples, woods, pagodas, and is worshipped and reviled. He is haunted by murderers or assassins, visited by witches and wizards, is tossed on the sea and beholds the monsters of the deep. He is again in bed and cold clammy corpses lie beside him, he is on the banks of the Nile and is kissed with "cancerous kisses" by the crocodiles. He smells horrible and pestilential odours and dwells in catacombs and charnel-houses. In a word, he experiences the most extraordinary sensations; sees, hears, smells, and feels the most bizarre and monstrous things possible or impossible; is beset by nameless and colossal and horrible "things," and undergoes the most wondrous transformations of personality.[63] His waking life in the intervals between such periods of intoxication is dull, melancholic, apathetic. Deprivation of the drug brings on the most frightful sufferings and torments, physical and mental. The unfortunate subject of this degraded habit soon becomes incapable of initiative or exertion. Recreation and games interest him but little. He becomes weak and tremulous, and has not energy for anything. His condition is weak, abject and pitiful until the next dose is taken. Then a startling transformation takes place. His energy returns, his interest revives, his disposition brightens, his spirits rise, he becomes energetic and capable, a living being once more. He can now transact business, work, write letters with a firm hand, study with profit and pleasure, walk with spirit even a ten-mile walk. And so it goes on day after day, each day his slavery to the drug becoming deeper. At times he makes, or is encouraged to make, an effort to abstain from the drug, but the suffering and agony that this entails is frightful. Now, not till now, he realises the full power of the thraldom in which he lives. "Deep as was his depression, wretched as was his state when the time for his customary dose arrived, the lapse of every subsequent hour increases his misery with such frightful rapidity and to such an appalling degree that the utmost limit of human endurance is reached."[64]

The evil effects of opium when given to children have been recently emphasised by Dr. T. D. Crothers, of Connecticut, U.S.A. The widespread use of proprietary medicines containing opium—e.g. many soothing syrups for children—has resulted in serious cerebral and mental degenerations, observed later in life and distinctly traceable to the opium.[65] Thus, in a family of four children of healthy parentage, as recorded by Dr. Crothers, one, a boy, was dull, passionate, and of low intellectual grade. He would over-eat and drink to excess anything he fancied. He was stupid and obstinate, and at times exceedingly irritable. No one in the family resembled him in any way, but all were healthy and vigorous in mind and body. The history of this boy was instructive. When one year old he had a convulsion, and was given opium daily for over a year, then at longer intervals, and finally it was abandoned. The parents noticed it was affecting him at the time, changing his mind and habits, and would not permit its use again. In a second healthy family one son became an impulsive inebriate at the age of eighteen years (the other children, brought up under the same surroundings, were temperate and normal). It transpired that this boy during infancy was treated with morphia for intestinal troubles. The drug was administered for a year by medical advice. He was dull and stupid during much of the time this drug was taken, and its use as a medicine was continued for some years after. Soon after the age of puberty the boy took to drink, and was now, at the age of eighteen, a periodic inebriate. Another case was that of a family in which two children suffered as the result of being nursed by a woman who was an opium-eater. One of these children, a girl, grew up a very nervous child, developed many hysterical disturbances, and at the close of adolescence (at twenty-four years of age) suddenly became addicted to opium and alcoholic excess, and showed much mental disturbance. Her brother, two years younger than herself, was nursed by the same woman, and was noted for his somnolence and general stupor during this time. He grew up and showed extreme nervousness and irritability. At one time he drank to excess, and was a gambler. He was changeable and became a clergyman, then a physician, and finally a speculator. He is now an opium-taker and neurasthenic, an invalid at thirty years of age. Two other children of the family, one born before the opium-eating woman was taken as nurse and the other after she was dismissed, were strong and healthy children, free from any peculiarities mental or bodily.

As regards treatment, isolation of the patient and supervision by one or two nurses, and gradual withdrawal of the drug (by diminution of the habitual dose) form the basis of treatment. In cases where through a mistaken or too rigorous *régime* the use of the drug has been abruptly and absolutely withheld a serious condition of collapse, capable of causing death, may result. The best treatment in such cases is to give morphia hypodermically in small doses, when the dangerous symptoms will disappear as if by magic, and then to gradually diminish the daily dose. Feeding should be carefully carried out, and the patient's strength kept up by the administration of ammonia and wine, with strychnine and arsenic as soon as possible (Richardson[66]). Insomnia should be overcome by sulphonal, or the bromides of sodium and ammonium. The cardiac condition should be improved by digitalis, strophanthus, and caffeine. Vomiting and diarrhœa must be treated as they arise. Baths and massage are valuable adjuvants to recovery. Hypnotic suggestion has been highly praised by some (Wettersbrand, Milne Bramwell), but to be effective it should be practised daily, and pushed to the extent of deep hypnosis at each sitting. Similarly, agencies that act by strongly impressing the feelings and imagination of the patient, such as profound emotional disturbance, religious ceremonies and pilgrimages, may act by way of suggestion towards cure.

[58] Talbot's Degeneracy: London, 1898. page 109. [59] Ibid. [60] The Opium Habit. [61] Review of Insanity and Nervous Disease, March, 1891. [62] Confessions of an English Opium-eater. [63] Most of these are drawn from the "Confessions" of De Quincey. [64] Mercier's "Vice, Crime, and Insanity," in Clifford Allbutt's System of Medicine, vol. viii. [65] Journal of the American Medical Association, May 19th, 1900. [66] The Asclepiad, 1884.

